# Rapidly Identifying New Coronavirus Mutations of Potential Concern in the Omicron Variant Using an Unsupervised Learning Strategy

**DOI:** 10.21203/rs.3.rs-1280819/v1

**Published:** 2022-02-25

**Authors:** Lue Ping Zhao, Terry Lybrand, Peter Gilbert, Thomas H. Payne, Chul-Woo Pyo, Daniel Geraghty, Keith Jerome

**Affiliations:** Fred Hutchinson Cancer Research Center; Vanderbilt University; Fred Hutchinson Cancer Research Center; University of Washington; Fred Hutchinson Cancer Research Center; Fred Hutchinson Cancer Research Center; Fred Hutchinson Cancer Research Center

**Keywords:** Omicron, haplotype, mutation, polymutant, SARS-CoV-2, single nucleotide variant, substitution, unsupervised machine learning

## Abstract

Extensive mutations in the Omicron spike protein appear to accelerate the transmission of SARS-CoV-2, and rapid infections increase the odds that additional mutants will emerge. To build an investigative framework, we have applied an unsupervised machine learning approach to 4296 Omicron viral genomes collected and deposited to GISAID as of December 14, 2021, and have identified a core haplotype of 28 polymutants (A67V, T95I, G339D, R346K, S371L, S373P, S375F, K417N, N440K, G446S, S477N, T478K, E484A, Q493R, G496S, Q498R, N501Y, Y505H, T547K, D614G, H655Y, N679K, P681H, N764K, K796Y, N856K, Q954H, N69K, L981F) in the spike protein and a separate core haplotype of 17 polymutants in non-spike genes: (K38, A1892) in nsp3, T492 in nsp4, (P132, V247, T280, S284) in 3C-like proteinase, I189 in nsp6, P323 in RNA-dependent RNA polymerase, I42 in Exonuclease, T9 in envelope protein, (D3, Q19, A63) in membrane glycoprotein, and (P13, R203, G204) in nucleocapsid phosphoprotein. Using these core haplotypes as reference, we have identified four newly emerging polymutants (R346, A701, I1081, N1192) in the spike protein (p-value=9.37*10^−4^, 1.0*10^−15^, 4.76*10^−7^ and 1.56*10^−4^, respectively), and five additional polymutants in non-spike genes (D343G in nucleocapsid phosphoprotein, V1069I in nsp3, V94A in nsp4, F694Y in the RNA-dependent RNA polymerase and L106L/F of ORF3a) that exhibit significant increasing trajectories (all p-values < 1.0*10^−15^). In the absence of relevant clinical data for these newly emerging mutations, it is important to monitor them closely. Two emerging mutations may be of particular concern: the N1192S mutation in spike protein locates in an extremely highly conserved region of all human coronaviruses that is integral to the viral fusion process, and the F694Y mutation in the RNA polymerase may induce conformational changes that could impact Remdesivir binding.

## Introduction

Since the first report of SARS-COVID2 in January 2020^1^, the virus has been continuously evolving with more mutations, periodically leading to major new variants that dominate local or global populations (https://www.cdc.gov/coronavirus/2019-ncov/variants). While many (Alpha, Beta, Gamma, Epsilon, Lota, Kappa, Mu, Zeta) are designated by the US CDC as variants being monitored (VBM), two have been designated variants of concern (VOC): Delta (B.1.617.2), rapidly becoming a dominant variant worldwide after its initial report in India (https://www.who.int/en/activities/tracking-SARS-CoV-2-variants/), and more recently the Omicron variant (B.1.1.529), which was first reported by scientists in South Africa and declared as a variant of concern (VOC) on November 26, 2021 by WHO (https://www.who.int/news/item/28-11-2021-update-on-omicron) and on November 30, 2021 by the US CDC (https://www.cdc.gov/media/releases/2021/s1201-omicron-variant.html). The primary impetus for this designation is that Omicron harbors many more mutations in spike protein than the Delta variant, and many of these mutations may impact transmissibility, disease severity, vaccine effectiveness, test reliability, and/or treatment effectiveness.

Compared with a handful of mutations in the Delta variant, Omicron is reported to have as many as 30 or more mutations in the surface glycoprotein, i.e., spike protein, although the actual number varies (https://www.who.int/news/item/26-11-2021-classification-of-omicron-(b.1.1.529)-sars-cov-2-variant-of-concern). Here we report preliminary results from profiling the mutational landscape of Omicron viruses to date (December 14, 2021), contrasting Omicron with the Delta and Alpha variants, identifying a set of core Omicron mutations in the spike protein as well as other viral proteins, and evaluating their potential structural and functional significance. We also identify a set of newly emerging mutants in the Omicron variant, several of which may be of particular concern.

## Mutation Landscape Of Omicron

As of December 14, 2021, a total of 4,296 Omicron sequences from 53 countries (see Supplementary Table S1) have been deposited to GISAID (https://www.gisaid.org/). Aligning these sequences with the first SARS-CoV-2 sequence (29,903 bp) allows identification of mutations/substitutions in every nucleotide of individual viruses (see Data and Methodology for data source, description, and processing). As an emerging variant, most nucleotides in the Omicron genomes are monomorphic, but 308 nucleotide sites have at least three observed mutations across 4,296 viruses. The mutated sites are referred to as single nucleotide variants (SNVs) in general, and further, if corresponding SNVs are non-synonymous in coding regions, are referred to as polymutants^2^. For the purpose of comparing Omicron with Delta and Alpha variants, we extend 308 SNVs to 837 SNVs, including SNVs of all three viral variants (see below). Effectively, aligned sequences, upon eliminating monomorphic nucleotides, can be transformed to a 4,296 by 837 matrix of mutation indicator 0’s or 1’s, corresponding to, respectively, the absence or presence of mutations.

**Figure 1:**
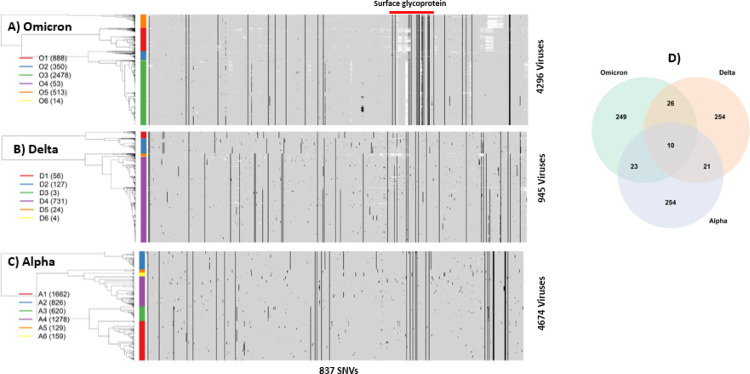
Mutation landscapes of A) Omicron, B) Delta and C) Alpha variants from profiling presence or absence of single nucleotide variants (SNVs) genome wide. Each mutation is colored in a black spot, wild type gray, and missing nucleotide white spot. The tree structure (on the left side of each heatmap) represents the similarities/dissimilarities among viruses. Colored bars correspond to clusters of viruses, which are labeled on the left side along with the number of viruses (in bracket). The surface glycoprotein (“Spike protein”) is highlighted by a red bar on top of the heatmap. D) Venn-diagram resulted from the overlapping analysis of identified single nucleotide variants (SNVs) from the Omicron, Delta and Alpha.

We apply unsupervised machine learning techniques to organize mutation indicators (see Data and Methodology for details). The technique hierarchically places similar rows of mutation indicators close together, and distant ones far apart. Figure 1A shows a heatmap representation of the Omicron mutation landscape, in which 0’s and 1’s are coded as gray and black, respectively. The white patches are due to missing data. Black vertical line(s) correspond to those mutations shared by most, if not all, Omicron viruses. Randomly distributed black dots represent sporadic mutations acquired by the Omicron variant. The hierarchical tree on the far-left side of the heatmap captures similarities/dissimilarities across these 4,296 viruses. These viruses are organized into six clusters (O1, O2, …, O6) color-coded by the color-bar on the left side to assist visual interpretation. The first cluster O1 (red bar) includes 888 viruses, the second cluster O2 (blue bar) includes 350 viruses, the third cluster O3 (green) includes 2478, and similarly for remaining three clusters (O4–6). Cross-tabulating clusters with continents suggests that most viruses in the O1 cluster are from Africa and Asia, and those in O3 from Europe and North America (Table S2).

To gain insights into Omicron in the context of other variants, we compared its landscape with those of Delta and Alpha variants, for which large numbers of sequences are available. We chose 945 Delta variant sequences and 4,675 Alpha variant sequences collected in Washington state (January 1 to Dec 3, 2021), most of which were sequenced by us with uniform high sequence quality. Following the same data processing protocol, their mutation landscapes were generated. The Delta variant originally emerged in India, and its enhanced transmissibility rapidly made it a dominant variant worldwide, including Washington state (https://www.doh.wa.gov/Emergencies/COVID19/Variants). Among all Delta viruses, there were as many as 1,480 SNVs with at least one mutation. To retain comparability with the Omicron landscape, we selected 311 sites as Delta-specific SNVs where the nucleotide-specific mutation percentage exceeded 0.5% and included these SNVs into the set of 837 SNVs shared with Omicron, Delta and Alpha variants (Fig. 1B). The Delta landscape has SNVs randomly distributed throughout the viral genome, and its mutation landscape is visually distinct from that of Omicron. Although Omicron and Delta variants share thirty-six SNVs (= 26 + 10), the majority of SNVs do not overlap (Fig. 1D).

Prior to the Delta wave, the Alpha variant was dominant, and was initially identified in the United Kingdom, rapidly spreading around the world^3^. Here we chose 4,674 sequences collected in the past year and found that 3,333 nucleotide sites had at least one mutation per site. Again, by imposing the same mutational threshold percentage of 0.5%, we identified 308 mutation sites and added them to the set of 837 SNVs. Figure 1C shows the Alpha mutation landscape, which includes many sporadic mutations but interestingly, three groups of clustered SNVs. Again, its landscape is distinct from the Omicron landscape, except for 33 overlapping SNVs (= 23 + 10 in Fig. 1D). Its landscape is also distinct from that of the Delta variant. The three variants share only ten SNVs.

## Profiles Of Spike Protein In Omicron, Delta, And Alpha

Much emphasis is placed on spike protein in many studies, because of its role in infection and relevance for vaccine development (the spike protein is indicated by red horizontal bars above the heatmap in Fig. 1A). To facilitate comparison, we selected 75 SNVs in the Spike protein, by combining SNVs from the Omicron, Delta and Alpha variants, and generated their corresponding heatmap representations, again demonstrating that there are more spike SNVs in the Omicron variant than in the Delta and Alpha variants (Figure S1A, B and C).

**Figure 2:**
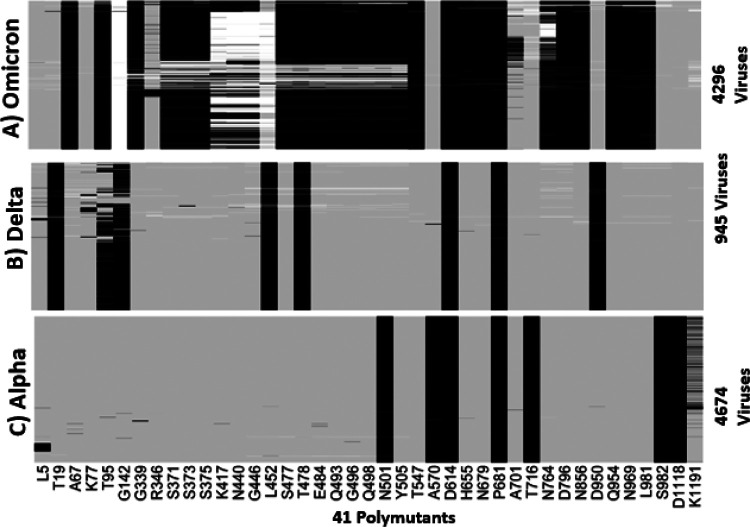
Profiles of 41 polymutants in spike protein (>5% mutation percentages) in A) Omicron, B) Delta and C) Alpha. Each mutation is represented by a black line, wildtype by a gray line, and missing data by a white line.

We translated nucleotides in spike protein to codons in the Omicron, Delta, and Alpha variants to generate protein sequences for comparison. We combined all polymutants from the three variants, resulting in a total of 371 polymutants, and profiled their landscapes. Despite a different visual representation, the message remains the same; Omicron has many more mutations in functional amino acids than Delta and Alpha variants (Figure S2A, B, C). To focus on more common polymutants (with mutation percentage exceeding 5%), we identified a total of 41 polymutants, shared across the three variants. Their mutation profiles are shown in Fig. 2, in which individual polymutants are labeled below the heatmap in Fig. 2C. Again, Omicron has many more polymutants than either Delta or Alpha. A total of 23 polymutants are unique to Omicron, while there are 4 overlapping polymutants (G142, T478, D614, T716) with the Delta variant and 3 overlapping polymutants (N501, D614, P681) with the Alpha variant. Note that we use a polymutant nomenclature without the ending amino acid designated, e.g., D614 as opposed to D614G, because several polymutants exhibit multiple mutations.

## Evolving Spike Polymutants Over Time

**Figure 3:**
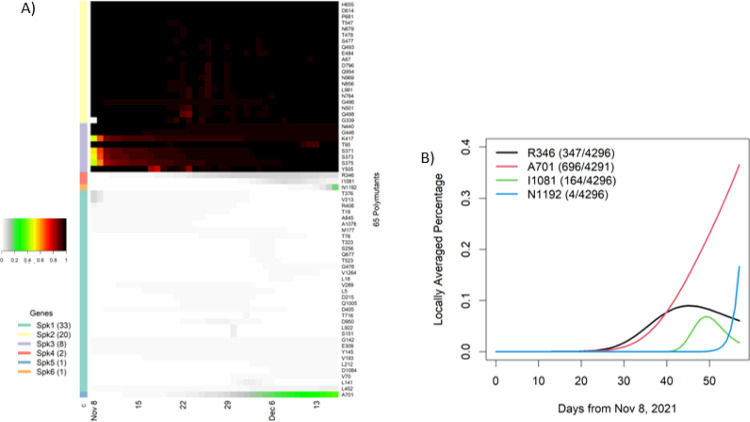
Temporal analysis of polymutants in spike protein: A) a heatmap representation of dynamic expansions/subtractions of 65 polymutants, in which locally averaged mutation percentages of every polymutant over time are shown in a row and is correspondingly colored from gray to dark red (color legend on the left side). By similarities/dissimilarities of their temporal patterns, all polymutants are clustered to six clusters (Spk1, …, Spk6), and numbers of polymutants in each cluster are indicated in bracket. B) Four polymutants with positive trajectories (in cluster Spk4, 5 and 6) are selected to illustrate their temporal patterns.

Omicron had a total of 65 polymutants, some of which had been consistently observed and others emerged over time. To gain insight into their temporal patterns, we fitted a non-parametric logistic regression model to each polymutant, which allowed us to estimate the locally averaged mutation percentage over time (see Data and Methodology section). These estimated daily mutation percentages were then collated into a matrix of 65 (polymutants) by 58 (days). Applying an unsupervised machine learning protocol, we organized these 65 polymutants by their temporal profiles into six clusters Spk1, 2, …, 6 (Fig. 3A). The cluster Spk2 included 20 polymutants that had averaged mutation probability of nearly one, as did the cluster Spk3 with 8 polymutants. As a result, the cluster analysis indicates that 28 polymutants (A67, T95, G339, S371, S373, S375, K417, N440, G446, S477, T478, E484, Q493, G496, Q498, N501, Y505, T547, D614, H655, N679, P681, N764, D796, N856, Q954, N969, L981) have been consistently present in nearly all Omicron sequences throughout time. Although absolute identification of an Omicron founder sequence may not be possible, it is nevertheless plausible that mutations at these 28 sites represents a core haplotype for the Omicron variant. To further support this conclusion, we evaluated haplotype frequencies, and found that 92% (3958/4296) of viruses carried a single haplotype VIDLPFNKSNKARSRYHKGYKHKYKHKF (Table 1). The next three most common haplotypes VIDLPFKNGNKARSRYHKGYKHKYKHKF, VIDLPFKKSNKARSRYHKGYKHKYKHKF and VIDSSSKKSNKARSRYHKGYKHKYKHKF were observed in only ~ 1% of all viruses. Given the ancestral haplotype (the reference haplotype from the original COVID sequence), it should be possible to identify other haplotypes that preceded the core haplotype listed in Table 1.

While most polymutants in the cluster Spk1 have sporadically low average mutation percentages, the remaining four polymutants (R346, A701, I1081, N1192) in Spk4–6 appear to have positive trajectories (Fig. 3A). From the non-parametric models, estimated locally averaged mutation percentages show that these four polymutants have significant non-linear upward trends (p-value = 9.37*10^− 4^, 1.0*10^− 15^, 4.76*10^− 7^ and 1.56*10^− 4^, respectively), shown in Fig. 3B. The polymutant R346K is observed among 347 of 4296 viruses and started increasing around day 25, leveling around day 40. This mutation has been reported in multiple countries (Table S3). Similarly, A701V and I1081V are also observed in multiple countries. The fourth polymutant N1192S emerged only very recently and has been reported from Hong Kong (2 sequences), Israel (1) and United Kingdom (1). Since this mutation is not reported from any African country thus far, one could postulate that this may be a new mutation originating outside of Africa.

## Evolving Polymutants In Non-spike Proteins

**Figure 4:**
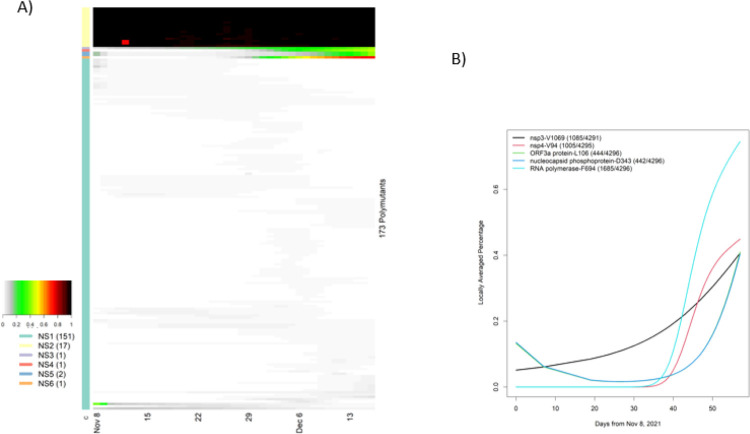
Temporal analysis of polymutants in non-spike protein genes, in which locally averaged mutation percentages of every polymutant over time are shown in a row and is correspondingly colored from gray to dark red (color legend on the left side). By similarities/dissimilarities of their temporal patterns, all polymutants are clustered to six clusters (NS1, …, NS6), and numbers of polymutants in each cluster are indicated in bracket. B) Six polymutants with positive trajectories (in cluster NS3, 4, 5 and 6) are selected to illustrate their temporal patterns.

SARS-CoV-2 has 10 genes, in addition to the spike protein (https://www.ncbi.nlm.nih.gov/sars-cov-2), and harbors many SNVs in both coding and non-coding regions. Given their potential roles in clinical disease, we translated nucleotides in these genes to codons. The ORF6 contained no polymutants. Nine other genes (ORF1ab, ORF3a, E, M, ORF7a, ORF7b, ORF8, N, ORF10) included one or more polymorphic sites, in which ORF stands for open reading frame, E envelope protein, M membrane glycoprotein and N nucleocapsid phosphoprotein. In total, there are 173 non-spike polymutants. Following the same temporal analysis protocol, we computed the locally averaged percentage of mutations daily. Applying the hierarchical clustering algorithm, we organized 173 temporal patterns into six clusters (NS1, NS2, …, NS6) (Fig. 4A). The cluster NS2 included 17 polymutants (yellow bar) with averaged mutation probability values of nearly one over time. Because these polymutants are present throughout time, they would naturally form a core haplotype of polymutants: (K38, A1892) of nsp3, T492 of nsp4, (P132, V247, T280, S284) of 3C-like proteinase, I189 of nsp6, P323 of RNA-dependent RNA polymerase (RdRp), I42 of Exonuclease, T9 of envelope protein, (D3, Q19, A63) of membrane glycoprotein, and (P13, R203, G204) of nucleocapsid phosphoprotein. Haplotype frequency analysis revealed that 96% of all Omicron viruses had this core haplotype (Table 2).

The 162 viruses in the cluster NS1 appeared to have relatively low mutation percentages and were not considered further; the remaining five polymutants (V1069 in nsp3, V94 in nsp4, F694 in RdRp, L106 in ORF3a and D343 in N) have positive trajectories, and L106 and D343 have nearly identical temporal patterns. To investigate these recently emerging polymutants closely, we extracted locally averaged mutation percentages and found that these polymutants are statistically significant, all with p-values less than 10^−15^ (Fig. 4B). Note that temporal curves for L106 and D343 are overlapping. Nearly synchronized concordance of both polymutants across different countries (Table S4) leads to the conclusion that these polymutants share the same haplotype. Given the positive trajectories of these five polymutants, it may be prudent to investigate their functional consequences and monitor their potential clinical correlations moving forward.

The core haplotypes of spike and non-spike genes enable us to construct a complete Omicron core haplotype, leveraging their high LD (shown in Table S5). Indeed, 91% of Omicron viruses exhibit the same complete core haplotype. Such a core haplotype should facilitate tracking transmission among individuals, movements of the virus, and possible mutations deviating from this Omicron core haplotype.

## Polymutant Haplotypes Of Emerging 9 New Mutations

As noted above, nine polymutants had positive trajectories as of December 14, 2021, i.e., (R346, A701, I1081, N1192) in the spike protein, V1069 in nsp3, V94 in nsp4, F694 in the RNA-dependent RNA polymerase, L106 in ORF3a, and D343 in nucleocapside phosphoprotein. To investigate how the Omicron core haplotype acquired these new mutations, we generated a haplotype profile for these polymutants and computed their frequencies (Table 3), where each haplotype was compared with the reference haplotype. Haplotypes were sorted according to the sample collection date. Since none of these new polymutants were present in the reference Omicron haplotype, most individuals (n = 1676) correspond to the reference haplotype, which is represented by an array of dots (highlighted in red). The first report date of the reference haplotype in the GISAID was October 24, 2021 in Easter Cap in South Africa. In total, 29 unique haplotypes have been observed, with varying numbers of mutations. To gain insights into their clade relationships, we computed distances between all haplotypes, and constructed a cladogram to depict relative relationship among haplotypes (Figure S3). As can be seen, four distinct clades can be identified.

Interestingly, the first reported haplotype was “RAINVVFGF” on October 17, 2021 in Abuja of Nigeria, Africa, indicating that mutants L106F and D343G were long in existence. To investigate this hapotype’s temporal and geographic profiles, we tabulated sample collection locations and dates (Table S6). After the first report, a virus with this haplotype was collected in Ontario, Canada on November 23, 2021. Afterwards, this polymutant haplotype was detected in multiple countries throughout the world, with recent surges of this haplotype from November 30 to December 7, mostly in Stockholm, Houston TX, England, Scotland, and Alberta CA. This same protocol could be used to trace every polymutant haplotype as part of a general monitoring protocol.

Among all new mutation haplotypes, probably the most worrisome is haplotype “RAVNVAFGY”, with 5 new mutations in a single Omicron core haplotype, each discussed in detail below. This mutated Omicron variant was first reported in England on December 12 and 60 cases were identified within 3–4 days. Investigating its spatial and temporal distribution, we noted that this mutation was present primarily in England from December 1 to 7, then appearing in Scotland and Wales on December 6 and 7, respectively (Table S7). It may be important to monitor this mutated Omicron haplotype closely, to assess its transmissibility and potential for severe disease.

## Structural Properties Of Spike Polymutants

We generated homology models to quickly assess the potential impacts of each of the 28 mutations identified in the core (“founding”) Omicron haplotype and identify mutations that might justify more detailed experimental characterization. A number of these positions also carry mutations in the earlier Alpha and Delta variants. Some, like D614G, are observed in all three variant families and the impact of this mutation has been well documented^4–8^. Other positions, such as P681, are also well documented as crucial for enhanced viral transmission. The Omicron variants thus far display the P681H mutation observed in earlier Alpha variants, unlike the P681R mutation observed in Delta, which has been documented experimentally to contribute to increased infectivity^9,10^.

**Figure 5:**
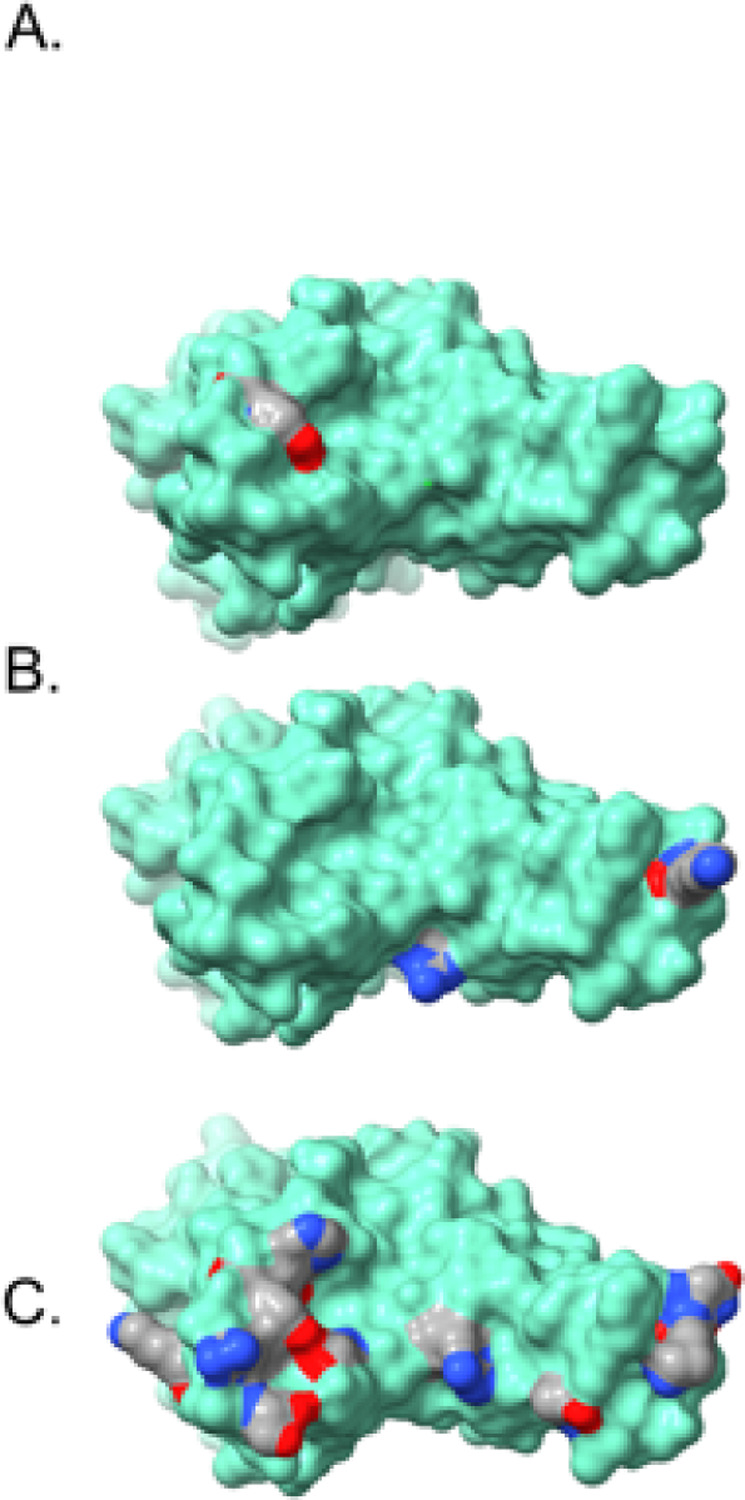
Receptor binding domain (RBD) contact surface observed in the angiotensin-converting enzyme 2 (ACE2) complex with the RBD (based on the crystal structure for the ACE2-RBD complex – PDB ID: 6M0J). Key RBD mutations that may impact ACE2 and/or antibody binding are color-coded by atom type (carbon – grey, nitrogen – blue, oxygen – red). A) Alpha variant B) Delta variant C) Omicron variant.

**Figure 6:**
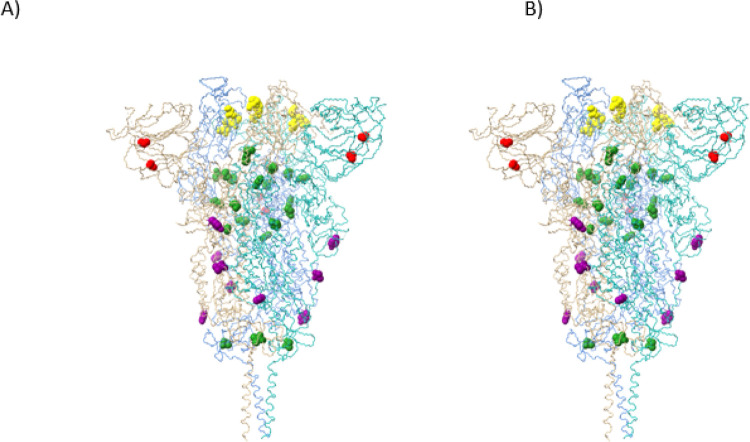
Stereo view of spike protein receptor trimer illustrating the locations of all Omicron spike protein mutations (excepting RBD contact surface mutations present in Figure 5). Monomer A is blue, monomer B is salmon and monomer C is aquamarine. All mutated residues are rendered as CPK models. Yellow residues (S371L, S373P, S375F, K417N) are located in the RBD where they may alter domain packing and/or monomer-monomer interactions. Green residues (T547K, N764K, N856K, Q954H, N969K, L981F, I1081V) are located at or near monomer-monomer interfaces in the receptor trimer assembly. Red residues (A67V, T95I) are located in the interior of the N-terminal domain. Purple residues (N655Y, A701V, D796Y) are located on the exterior surface of the trimer assembly in regions that are potential antibody binding sites. The homology model is based on reference spike protein trimer structure PDB ID: 7KRS.

The Omicron spike protein mutations can be classified generally into four sub-categories. Unsurprisingly, most mutations appear in the receptor binding domain (RBD). Most of these mutations (G339D, N440K, G446S, S477N, T478K, E484A, Q493R, G496S, Q498R, N501Y, Y505H) present on the RBD surface (Fig. 5), and it is reasonable to assume that these mutations may impact angiotensin-converting enzyme 2 (ACE2) receptor binding and/or recognition and binding by neutralizing antibodies (nAbs). If the Omicron variant originated in an immune-compromised patient with a prolonged infection, as has been speculated^2^, this latter scenario seems quite plausible. A smaller set of RBD mutations (S371L, S373P, S375F, K417N) display little or no surface exposure (Fig. 6), but the location and non-conservative nature of these mutations suggest that they may affect local conformation, in particular monomer-monomer contacts in the receptor trimer, and thus impact ACE2 and/or nAb binding indirectly via mutation-induced conformational changes.

A third set of mutations (T547K, N764K, N856K, Q954H, N969K, L981F) occur in the interior of the receptor trimer assembly at positions that likely will alter monomer-monomer packing interactions (Fig. 6), and these mutations may well affect overall receptor structure and/or dynamics and flexibility. Similarly, there are two mutations (A67V, T95I) in the interior of the N-terminal domain (Fig. 6). These mutations likely alter side chain packing interactions and may affect the domain conformation. This may be significant, as a number of neutralizing antibodies appear to bind sites in this domain. Two additional mutations (N655Y, D796Y) are present on the surface of the receptor trimer stalk region (Fig. 6) where they may be potential contact residues for nAbs. Finally, there are two mutations (N679K, P681H) in the S1/S2 cleavage segment. As noted above, mutations in this region have been shown previously to enhance spike protein cleavage, thus leading to increased infectivity.

In addition to these mutations in the “founding” Omicron variant, our unsupervised learning strategy revealed nine mutations in the viral genome that are emerging and possibly becoming established. In earlier studies, we have shown that our method can identify significant mutations long before they are characterized clinically. For example, we observed the emergence of the P681R mutation in the U.S. several months before the Delta variant became dominant^11^. We have therefore used homology modeling studies to assess the potential impact of these newly emerging mutations.

Four mutations occur in the spike protein. The R346K mutation is in the RBD (Fig. 5C); while an arginine to lysine mutation is often classified as a conservative mutation with little structural or functional consequence, there are situations where the two residues are not readily interchangeable, due to the distinct chemistry of the arginine guanidinium group (hydrogen bonding patterns, π-system interactions, etc.) compared to the primary amine group present in the lysine side chain. As a result, the R346K mutation might impact receptor and/or nAb recognition and binding. The A701V mutation is located on the exterior of the receptor trimer stalk region (Fig. 6), where it could impact nAb binding, much like N655Y and D796Y discussed above. The I1081V mutation is in the interior of the receptor trimer assembly and might influence conformation via altered side chain packing interactions. However, both A701V and I1081V are conservative mutations and may have little impact.

**Figure 7:**
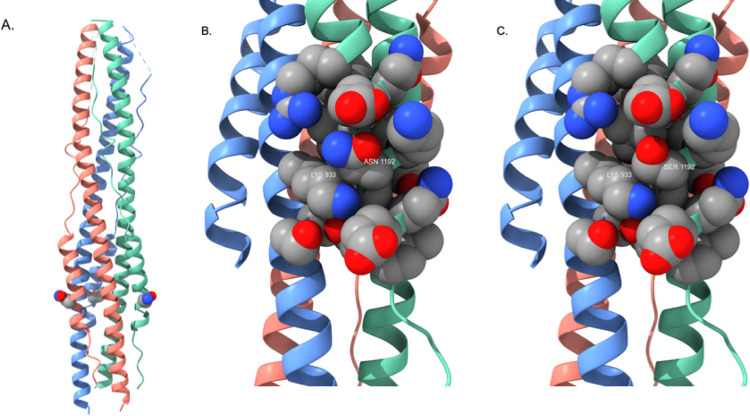
A) Post-fusion six-helix bundle (PDB ID: 6LXT) illustrating the location of the N1192S mutation. S1192 is rendered as a CPK residue (one per receptor monomer) and color-coded by atom type as described in Figure 5. B) Detailed view of side chain packing interactions formed by N1192. K933 is from the neighboring monomer; this image illustrates the tight packing contacts formed by proximate side chains that stabilize the six-helix bundle fusion structure. C) Detailed view of side chain packing interactions formed by the S1192 mutant. The helix bundle orientation is identical to that in Figure 7B.

The fourth emerging spike protein mutation, N1192S, is less prevalent thus far but is extremely interesting and of potential concern. This mutation is located in the heptapeptide repeat sequence 2 (HR2). This region is highly conserved across all human coronaviruses and is a key component in the formation of a six-helix bundle with HR1 segments to facilitate the fusion process with target host cells^12^. As a result, this region of the spike protein has been the focus of ongoing attempts to develop “universal” fusion inhibitors for therapeutic application in coronavirus infections. A crystal structure of the post-fusion six-helix bundle formed by HR1 and HR2 segments in the spike protein trimer is available^13^, enabling visualization of the N1192S mutation site (Fig. 7). It is not clear from these simple homology modeling exercises what the full impact of this mutation might be on packing interactions in the six-helix bundle. However, detailed views of the mutation site in Figs. 7B and 7C that display specific helix-helix contacts (K933 from one monomer with position 1192 in the neighboring monomer) imply that packing interactions between monomer units in the bundle will be altered, so further experimental study may be warranted to determine what effect this mutation might have on viral fusion efficiency with host cells.

Three emerging mutations are identified in the ORF1ab gene. One mutation, V1069I, maps to a region in the non-structural protein 3 (nsp3), just prior to the nsp3 nucleic acid binding domain, but there is no relevant structural information for this region of nsp3 to assess possible impacts. A second mutation, V94A, maps to a region located between putative transmembrane helices 1 and 2 in the nsp4 protein, and there is likewise no relevant experimental structural data for this region of nsp4.

**Figure 8:**
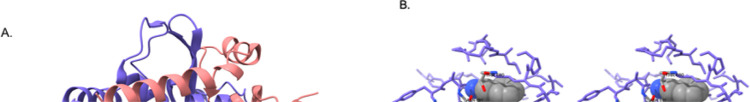
A) RNA-dependent RNA polymerase (blue) complex formed with non-structural protein 7 (green) and non-structural protein 8 (salmon). Primer and template RNA strands (lime green) are depicted as ribbons extending from the active site. The mutated residue (F694Y) is rendered as a red CPK model. B) Stereo view of active site with bound Remdesivir molecule (ball-and-stick rendering). F694 is rendered as a CPK model, and active site residues in direct contact with the inhibitor molecule are labeled. All atoms are color-coded by atom type as described in Figure 5. C) Stereo view of active with bound Remdesivir in the F694Y mutant. The orientation, labeling and color-coding are identical to that in Figure 8B. The mutant homology model has not been subjected to further structural refinement and this image clearly displays steric clashes generated by substitution of the tyrosine side chain. Models in panels A, B and C are all based on the cryo-electron microscopy structure PDB ID: 7BV2.

A third mutation in ORF1ab, F694Y, occurs in the RNA-dependent RNA polymerase and is most intriguing. Figure 4B illustrates the dramatic increase in this mutation since 8 December 2021, suggesting that F694Y may provide a notable fitness advantage for the virus. The location of this mutation in the polymerase is show in Fig. 8A, and Fig. 8B highlights the close proximity of the mutation to key residues involved in RNA binding^14^. Of greater potential significance, the mutation site interacts directly with several residues involved in Remdesivir binding^14^, as shown in Fig. 8C. While the phenylalanine to tyrosine mutation is a relatively conservative change, tyrosine is larger and introduces a hydrogen bond donor/acceptor at this position, so the mutation will undoubtedly induce at least some subtle conformational changes in this region of the substrate and inhibitor binding site, changes that may well alter Remdesivir binding and impact its therapeutic effectiveness. This general pattern, namely mutation of a residue near the inhibitor binding/active site, rather than mutation of key active site residues directly, is a commonly observed drug resistance development mechanism in target enzymes of many pathogens (e.g., inhibitor resistance development in HIV protease often exhibits this profile). Given the possible implications of this mutation, prompt experimental investigation seems justified.

The L106F mutation occurs in ORF3a, in a region that codes for a putative ion channel 3a^15^. This mutation occurs near the amino terminus of helix 3 on the exterior face. This residue is presumably exposed to the lipid environment, so it is unclear how it may affect function, if at all. However, it is well established that mutations in ion channel 3a can impact ion conductance and viral viability^15^.

The final emerging mutation, D343G, is present in the nucleocapsid phosphoprotein C-terminal dimerization domain, located in a short loop connecting b-strand b2 to the a6 helix. This residue is not directly involved in any dimer contacts, and its relatively exposed position in a flexible loop region makes it difficult to anticipate the structural consequences. It is certainly true that the glycine substitution at this position should increase loop flexibility, and this might facilitate domain conformational rearrangement necessary for the dimerization process. This may impart a fitness advantage for the virus, as experimental studies suggest that the nucleocapsid homodimer is the stable form in soluble, and that the homodimer can bind short ssRNA molecules without any ancillary proteins^16^.

## Discussion

We present all major mutations currently reported in the spike protein of the Omicron variant, as well as those occurring in non-spike genes, as an Omicron “mutation landscape”. The Omicron landscape is clearly distinct from the mutation landscapes of the Delta and Alpha variants. We observe twenty-eight polymutants in the spike protein and seventeen polymutants in the non-spike genes that we define as core haplotypes. These two core haplotypes form a complete Omicron haplotype, observed in 91% of reported Omicron sequences. Our unsupervised learning strategy reveals that the Omicron variant is now acquiring four new mutations in the spike protein (R346K, A701V, I1081V, N1192S) and five new mutations in nsp3 (V1069I), nsp4 (V94A), the RNA-dependent RNA polymerase (F694Y), ORF3a (L106F) and nucleocapsid phosphoprotein (D343G). This observation suggests that Omicron may be mutating more rapidly, and that close monitoring, using a protocol such as that presented here, will be essential to make appropriate public health decisions and help guide future vaccine design efforts. Two of these newly emerging mutations are particularly worrisome. The N1192S mutation is located in a spike protein region, the HR2 sequence, that exhibits extremely high conservation across all human coronavirus, presumably due to the importance of this region in the host cell fusion process. If this mutation persists and expands, it may indicate that the virus is evolving an even more efficient fusion capability that could lead to a further enhancement of infectivity. The F694Y mutation in the polymerase enzyme occurs at a position that seems likely to impact binding of Remdesivir and related viral inhibitors. It would be most interesting to attempt to correlate clinical records with the rapid and dramatic emergence of this mutation, to explore whether it is appearing primarily in patients who have been treated with Remdesivir. Prompt and detailed study of this new mutation seems advisable.

Another major concern is whether the Omicron variant may lead to severe disease and thus may increase hospitalization risk. While early clinical reports are encouraging, the recent emergence of the Omicron variant and lack of robust clinical surveillance programs for newly emerging variants means that much critical information is still not readily available. We have attempted to address this issue in our earlier work^17^. We discovered and replicated an association of SNV-haplotypes (t19839-g28881-g28882-g28883) with hospitalization risk among COVID positive patients, in which the mutated haplotype “caac” had a significant association with hospitalization risk (OR = 5.46, p = 4.71*10^−12^ in the replication cohort). Fortunately, this haplotype is absent in current Omicron patients to date. However, it is nevertheless worrisome, since the most common haplotype in current Omicron patients is “taac” (99%). If this single mutation at t19839 emerges in Omicron variants, the resulting mutant might create a “perfect storm”, exhibiting extremely high transmissibility and elevated disease severity. This observation underscores the importance of active vaccination to stop the continuing spread, and resultant mutation, of the virus, as well as the need for active surveillance for clinical relevance of emerging variants.

Identifying core Omicron haplotypes also permit us to apply next generation sequence-based genotyping (NGS) to focus on specific sets of SNVs at very low cost, with a protocol modified from sequencing the entire viral genome^17^. NGS sequencing can be performed at hospital sites as a routine, integrated with existing genotyping services supporting other activities, in much the same way as HLA typing and chimerism testing is done routinely in support of hematopoietic cell transplants, thus enabling more widespread and continuous monitoring for specific Omicron and future variants. Local monitoring could also better facilitate direct geographic reporting and connecting genetic data with clinical outcomes as discussed below.

Genomic surveillance has been enormously helpful for us to identify emerging new variants^18,19^, but has also resulted in great uncertainty when there is no relevant clinical data that can be correlated with the new variants. To close this knowledge gap, we propose expanding current genomic surveillance to include electronically captured health records, e.g., discharge summary records from hospitalized patients, abbreviated medical charts from clinical visits, vaccination status reports, vital statistics reports, and clinical/demographic information recorded on COVID test requests. Given the rapid evolution of variants and their potential implications for public health, we should explore ways for data from hospitalized and ambulatory patients to be available for this analysis sooner than is currently possible. Balancing individuals’ privacy rights, the expense of gathering relevant data and the time-sensitive nature of the relevant clinical data, we propose to gather a minimum set of clinical and demographic variables from patients whose viral genomes have been sequenced and who have consented to the expanded surveillance program. We understand that knowledge of clinical manifestations of COVID is evolving rapidly, so the specific clinical data necessary to discover correlations between viral mutations and clinical impact may also need to evolve rapidly. Analytic methods make larger amounts of data practical to use than has been true historically, for example, entire sets of notes, imaging and pathology reports, and laboratory results. This surveillance program could help continuously monitor all CDC-designated variants and their evolving mutations, as well as aid correlation of evolving variants with clinical outcomes among vaccinated and non-vaccinated individuals. This surveillance program should help us to assess associations of known variants with vaccine evasion and short-term clinical disease in real time, and to discover novel variants that may evade vaccination, or may impact on clinical disease (short and long-term).

The COVID pandemic is unprecedented in recent history and continues to present consider challenges for clinical treatment delivery and public health policy. The recently implemented genomic surveillance effort has been instrumental to enable discovery of new variants and to inform society at large in a timely fashion. However, current experience with the Omicron variant suggests the need to expand and better coordinate clinical data collection with genomic surveillance and biological studies. It is time for us to “get ahead” of a next COVID variant wave.

## Data And Methodology

### Selected viral sequences

We searched GISAID to retrieve viral sequences of Omicron, Delta and Alpha variants used in this study on December 3, 2021 via the web portal https://www.gisaid.org^20^. All sequences had complete and high coverage. Using the same search criteria, we retrieved metadata associated with all individual viruses. With respect to Omicron (B.1.1.529), we included all Omicron sequences deposited to GISAID by the search date. Viruses were collected between November 8 to December 1, 2021 worldwide.

For comparison purpose, we selected sequence data of the Delta variant (B.1.617.2), which was first reported in India and remained as the most observed viruses throughout the world. Because of abundant sequences available, we chose to focus on 945 viral sequences contributed from Washington state, most of which were sequenced by us at University of Washington under contract to Washington state and CDC. Viruses were collected from April 3 to November 23, 2021.

To place the comparison in the context of evolving variants, we further included sequence data of the Alpha variant (B.1.1.7), which was the first major variant and initially reported in United Kingdom, gradually spreading to Europe and the rest of the world. Again, a large number of viruses were sequenced and deposited to GISAID. To focus our analytics, we downloaded 4674 viral sequences deposited from Washington state. Viruses were collected from January 19 to September 8, 2021.

Following the data access guideline at GISAID, we were able to download viral sequences and associated metadata without any issues.

### Sequence processing and transformation

All deposited sequences to GISAID have been subject to systematic quality control, especially those with complete and high coverage. Treating the first SARS-CoV-2 genomic sequence as the reference sequence^21^, we performed whole genome sequence alignment, using MAFFT^22^, which yielded a complete “rectangular nucleotide sequence matrix” of nucleotides by viruses. While majority nucleotides were monomorphic, a small fraction of nucleotides had at least one mutation or substitution, in comparison with the reference sequence. A nucleotide with at least one observed mutation is referred to as single nucleotide variant (SNV). When such a SNV located in a gene and was also non-synonymous, it could be equivalently referred to as a polymutant. To facilitate unsupervised learning, we transformed the matrix of nucleotides to a matrix of binary indicators for the presence/absence of mutations. Monomorphic nucleotides were transformed to columns of zeros and were eliminated from further analysis. Note that genomewide alignment inevitably may miss structural mutations (insertions, deletions, or local rearrangements). Commonly, such structural mutations could impact on the performance of amplicons used in the sequencing reagents and hence on sequence assembly. Indeed, blocks of missing nucleotides (white spaces in heatmap, Fig. 1) potentially suggest such structural mutations. While separate effort is required for investigating structural mutations, their presence does not affect the validity of the current statistical analysis, relying on multiple sequences.

### Unsupervised learning

Following the sequence processing and transformation, each viral sequence can now be represented by a vector of 0’s and 1’s, corresponding to absence and presence of a mutation respectively. In absence of meaningful clinical parameters, we proposed to use unsupervised learning approaches to organize and explore this matrix of SNVs, using a statistical learning strategy we described previously^11^. In this project, the hierarchical clustering method was applied to organize this matrix: the first step was to compute distances between pairs of viral mutation indicators based on the Yule similarity measure^23^, and the second step was to hierarchically organize all viruses so that similar row vectors were clustered closer based on the Ward algorithm^24^. The matrix of organized mutation indicators was then displayed in the heatmap. To facilitate the data interpretation, we chose to cluster all samples to six groups (K1, K2, …, K6), which were color-labeled as a vertical sidebar.

### Haplotype analysis and missing nucleotide imputation

Multiple SNVs obtained from same individuals were generally from a single clone of viruses, and their nucleotides thus formed a single haplotype, referred to as a SNV-haplotype. Due to recent evolutionary history, most SNVs were in high linkage-disequilibrium (LD), and such haplotypes were broken down usually by emerging variants. Hence, haplotypes were informative for evolving viral variants. Due to high LD, the number of haplotypes was typically smaller than the theoretic expectations with multiple SNVs. Hence, focusing on haplotypes facilitated parsimonious investigation of otherwise complex RNA sequences or many individual SNVs.

The high LD among SNVs was also helpful for imputing missing nucleotides. Due to nature of sequencing technologies, a small fraction of nucleotides was untyped, and were coded as “n”. Given high linkage disequilibrium across all SNVs, we assembled a panel of polymorphic nucleotides that had no missing values and had not been selected into SNVs of interest and treated them as an “imputation base”. Fusing on one SNV with nucleotides in the imputation base, we computed their haplotype frequencies, and used their haplotype frequencies to compute posterior probabilities to impute missing nucleotides, in the same way as imputing single nucleotide polymorphisms^25^.

### Non-parametric regression

A polymutant may star with a mutated amino acid or acquire a new mutation across multiple viruses over time. To characterize its temporal pattern, we apply a generalized additive model (GAM) to fit a non-linear logistic model, regressing mutation indicators over viral collection times^26,27^. After fitting the model, we computed the fitted values as locally averaged mutation percentage daily to characterize the temporal patterns of mutation occurrences. The same technique is applicable to compute average number of unique polymutants daily.

### Structural modeling

All model building and analysis was performed using the ChimeraX interactive molecular graphics package^28^. Protein Data Bank (PDB) structures 6ZWV, 7KRR, and 7KRS were used as templates for homology modeling of spike protein, structure 6M0J was used as template for spike protein receptor binding domain contact surface models, structure 6LXT was used to model the post-fusion six-helix bundle, and structure 7BV2 was used for modeling of the RNA-dependent RNA polymerase complex. Mutated side chain conformations were generated using the backbone-dependent rotamer library of Shapovalov and Dunbrack^29^ and selected to minimize steric clash with neighboring residues. No additional structural refinement was performed.

## Supplementary Material

Supplement 1

Supplement 2

## Figures and Tables

**Table 1.** Haplotype frequencies of 28 polymutants (A67V, T95I, G339D, S371L, S373P, S375F, K417N, N440K, G446S, S477N, T478K, E484A, Q493R, G496S, Q498R, N501Y, Y505H, T547K, D614G, H655Y, N679K, P681H, N764K, K796Y, N856K, Q954H, N69K, L981F), observed among all Omicron viruses.

Polymutant Haplotypes	Freq	A67	T95	G339	S371	S375	K417	N440	G446	S477	T478	E484	Q493	G496	Q498	N501	Y505	T547	D614	H655	N679	P681	N764	N764	D796	N856	Q954	N969	L981
	Mutant	V	I	D	L	P	F	N	K	S	N	K	A	R	S	R	Y	H	K	G	Y	K	H	K	Y	K	H	K	F
VIDLPFNKSNKARSRYHKGYKHKYKHKF	3958	.	.	.	.	.	.	.	.	.	.	.	.	.	.	.	.	.	.	.	.	.	.	.	.	.	.	.	.
VIDLPFKNGNKARSRYHKGYKHKYKHKF	45	.	.	.	.	.	.	K	N	G	.	.	.	.	.	.	.	.	.	.	.	.	.	.	.	.	.	.	.
VIDLPFKKSNKARSRYHKGYKHKYKHKF	36	.	.	.	.	.	.	K	.	.	.	.	.	.	.	.	.	.	.	.	.	.	.	.	.	.	.	.	.
VIDSSSKKSNKARSRYHKGYKHKYKHKF	24	.	.	.	S	S	S	K	.	.	.	.	.	.	.	.	.	.	.	.	.	.	.	.	.	.	.	.	.
VIDLPFKNGNKARSRYHKGYKHNYKHKF	8	.	.	.	.	.	.	K	N	G	.	.	.	.	.	.	.	.	.	.	.	.	.	N	.	.	.	.	.
VIDLPFNKSNKARSRYHKGYKHNYKHKF	8	.	.	.	.	.	.	.	.	.	.	.	.	.	.	.	.	.	.	.	.	.	.	N	.	.	.	.	.
VIDLPSNKSNKARSRYYKGYKHKYKHKF	8	.	.	.	.	.	S	.	.	.	.	.	.	.	.	.	.	Y	.	.	.	.	.	.	.	.	.	.	.
VIDFPFNKSNKARGRYHTGYKHKYNHKL	6	.	.	.	F	.	.	.	.	.	.	.	.	.	G	.	.	.	T	.	.	.	.	.	.	N	.	.	L
VIDLPFNKSNKARSRYHKGYKHNDNQNL	6	.	.	.	.	.	.	.	.	.	.	.	.	.	.	.	.	.	.	.	.	.	.	N	D	N	Q	N	L
VIGLPFNKSNKARSRYHKGYKHKYKHKF	6	.	.	G	.	.	.	.	.	.	.	.	.	.	.	.	.	.	.	.	.	.	.	.	.	.	.	.	.
VIDLPFNKSNKVRSRYHKGYKHKYKHKF	5	.	.	.	.	.	.	.	.	.	.	.	V	.	.	.	.	.	.	.	.	.	.	.	.	.	.	.	.
VIDLPFNNSNKARSRYHKGYKHKYKHKF	5	.	.	.	.	.	.	.	N	.	.	.	.	.	.	.	.	.	.	.	.	.	.	.	.	.	.	.	.
VIDLPSNKSNKARSRYHKGYKHKYKHKF	5	.	.	.	.	.	S	.	.	.	.	.	.	.	.	.	.	.	.	.	.	.	.	.	.	.	.	.	.
VIDSSSNKSNKARSRYHKGYKHKYKHKF	5	.	.	.	S	S	S	.	.	.	.	.	.	.	.	.	.	.	.	.	.	.	.	.	.	.	.	.	.
VTDLPFNKSNKARSRYHKGYKHKYKHKF	5	.	T	.	.	.	.	.	.	.	.	.	.	.	.	.	.	.	.	.	.	.	.	.	.	.	.	.	.
All rare haplotypes (<5)*	166	.	.	.	.	.	.	.	.	.	.	.	.	.	.	.	.	.	.	.	.	.	.	.	.	.	.	.	.

* Full list of all haplolypes are given in the Supplementary materials

**Table 2.** Haplotype frequencies of 17 polymutants (K38A, 1892) of nsp3, T492 of nsp4, (P132, V247, T280, S284) of 3C-like proteinase, I189 of nsp6, P323 of RdRp, I42 of Exonuclease, T9 of envelope protein, (D3, Q19, A63) of membrane glycoprotein, (P13, R203, G204) of nucleocapsid phosphoprotein, all of which locate outside of the spike gene, observed among all Omicron viruses.

Polymutant Haplotype	Frequency	nsp3.K38	nsp3-A1892	nsp4-T492	nsp5-P132	nsp5-V247	nsp5-T280	nsp5-S284	nsp6-I189	RdRp-P323	nsp14-I42	E-T9	M-D3	M-Q19	M-A63	N-P13	N-R203	N-G204
	Mutant	R	T	I	H	V	T	S	V	L	V	I	G	E	T	L	K	R
RTIHVTSVLVIGETLKR	4131	.	.	.	.	.	.	.	.	.	.	.	.	.	.	.	.	.
RTIHVTSVLVIDETLKR	52	.	.	.	.	.	.	.	.	.	.	.	D	.	.	.	.	.
RTIHVTSVLVIGETPKR	18	.	.	.	.	.	.	.	.	.	.	.	.	.	.	P	.	.
KAIHVTSILVIDETLKR	13	K	A	.	.	.	.	.	I	.	.	.	D	.	.	.	.	.
RTIHVTSVLVIGEALKR	11	.	.	.	.	.	.	.	.	.	.	.	.	.	A	.	.	.
RTIPVTSVLVIDETLKR	6	.	.	.	P	.	.	.	.	.	.	.	D	.	.	.	.	.
RTIHVTSVLVIDQALKR	5	.	.	.	.	.	.	.	.	.	.	.	D	Q	A	.	.	.
RTIYVTSVLVIGETLKR	5	.	.	.	Y	.	.	.	.	.	.	.	.	.	.	.	.	.
rare haplotypes (<5)	55	.	.	.	.	.	.	.	.	.	.	.	.	.	.	.	.	.

**Table 3.** Haplotype analysis of 9 emerging mutations in Omicron viruses; haplotype frequencies, changes of individual amino acids from the reference, number of observed mutations in the haplotype, date and location of the first report. Note that all haplotypes are sorted by time and location of the first report. The reference haplotype is highlighted in red font.

ID	Emerging Haplotype	Freq	S-R346	S-A701	S-I1081	S-N1192	nsp3-V1069	nsp4-V94	RdRp-F694	ORF3a-L106	N-D343		First Reported	
		Ref	R	A	I	N	V	V	F	L	D	#	Date	Locations
1	RAINVVFGF	148	.	.	.	.	.	.	.	F	G	2	2021-10-17	Africa/Nigeria/Abuja
2	RAINVVLDF	1676	.	.	.	.	.	.	.	.	.	0	2021-10-24	Africa/South Africa/Eastern Cape
3	RVINIVLDF	154	.	V	.	.	I	.	.	.	.	2	2021-11-10	Africa/South Africa/Gauteng
4	RAINIVLDF	344	.	.	.	.	I	.	.	.	.	1	2021-11-11	Africa/Botswana/South East/Greater Gaborone/Gaborone
5	KAINVVLDF	233	K	.	.	.	.	.	.	.	.	1	2021-11-17	Africa/South Africa/Gauteng/Tshwane
6	RAINIVLDY	27	.	.	.	.	I	.	Y	.	.	2	2021-11-20	Europe/United Kingdom/England
7	RAINVALDY	339	.	.	.	.	.	A	Y	.	.	2	2021-11-23	Europe/United Kingdom/Scotland
8	RAINVVLDY	390	.	.	.	.	.	.	Y	.	.	1	2021-11-23	Europe/United Kingdom/Scotland
9	RVINIALDY	463	.	V	.	.	I	A	Y	.	.	4	2021-11-24	Europe/United Kingdom/England
10	KAINVVLDY	57	K	.	.	.	.	.	Y	.	.	2	2021-11-24	Europe/United Kingdom/England
11	RAINVAFGY	53	.	.	.	.	.	A	Y	F	G	4	2021-11-25	Europe/United Kingdom/England
12	RAVNWFGF	35	.	.	V	.	.	.	.	F	H	3	2021-11-25	Africa/Senegal/Dakar/IRESSEF DIAMNIADIO
13	RVINVVLDF	2	.	V	.	.	.	.	.	.	.	1	2021-11-25	Africa/South Africa/Western Cape
14	RAINVVLGF	1	.	.	.	.	.	.	.	.	G	1	2021-11-25	North America/Canada/Ontario
15	RVINIVLDY	72	.	V	.	.	I	.	Y	.	.	3	2021-11-26	Europe/United Kingdom/England
16	KAINVALDY	55	K	.	.	.	.	A	Y	.	.	3	2021-11-27	Europe/United Kingdom/England
17	RAINIALDY	19	.	.	.	.	I	A	Y	.	.	3	2021-11-27	Europe/United Kingdom/England
18	RAINVVFGY	75	.	.	.	.	.	.	Y	F	G	3	2021-11-28	Europe/United Kingdom/England
19	KAINVVFGF	1	K	.	.	.	.	.	.	F	G	3	2021-11-29	North America/USA/Alaska/Anchorage-Mat Su
20	RAVNWFGY	68	.	.	V	.	.	.	Y	F	G	4	2021-11-30	Europe/United Kingdom/England
21	RAVNVAFGY	60	.	.	V	.	.	A	Y	F	G	5	2021-12-01	Europe/United Kingdom/England
22	RAINVALDF	7	.	.	.	.	.	A	.	.	.	1	2021-12-01	Europe/United Kingdom/England
23	RAINVVFDF	3	.	.	.	.	.	.	.	F	.	1	2021-12-04	North America/USA/Texas/Houston County
24	RVINIALDF	1	.	V	.	.	I	A	.	.	.	3	2021-12-05	Europe/United Kingdom/England
25	RVISIVLDF	3	.	V	.	S	I	.	.	.	.	3	2021-12-06	Asia/Israel
26	RAINIALDF	1	.	.	.	.	I	A	.	.	.	2	2021-12-06	Europe/United Kingdom/England
27	RVISIALDY	1	.	V	.	S	I	A	Y	.	.	5	2021-12-07	Europe/United Kingdom/England
28	RAVNVAFGF	1	.	.	V	.	.	A	.	F	G	4	2021-12-07	Europe/United Kingdom/England
29	KAINVALDF	1	K	.	.	.	.	A	.	.	.	2	2021-12-07	Europe/United Kingdom/England

# number of mutations observed on the polymutant halotype

## Data Availability

All sequence data analyzed here are publicly available at GSIAD (https://www.gisaid.org/).
