# Supplementary material for: Rapidly Identifying New Coronavirus Mutations of Potential Concern in the Omicron Variant Using an Unsupervised Learning Strategy
Source: Res Sq. 2022 Feb 25:rs.3.rs-1280819. Preprint. [Version 1] doi: 10.21203/rs.3.rs-1280819/v1 (PMC8887078; doi:10.21203/rs.3.rs-1280819/v1)
Supplement: Supplement 1 [file 024ea114269a684336b240a4.pptx]

## Slide 1
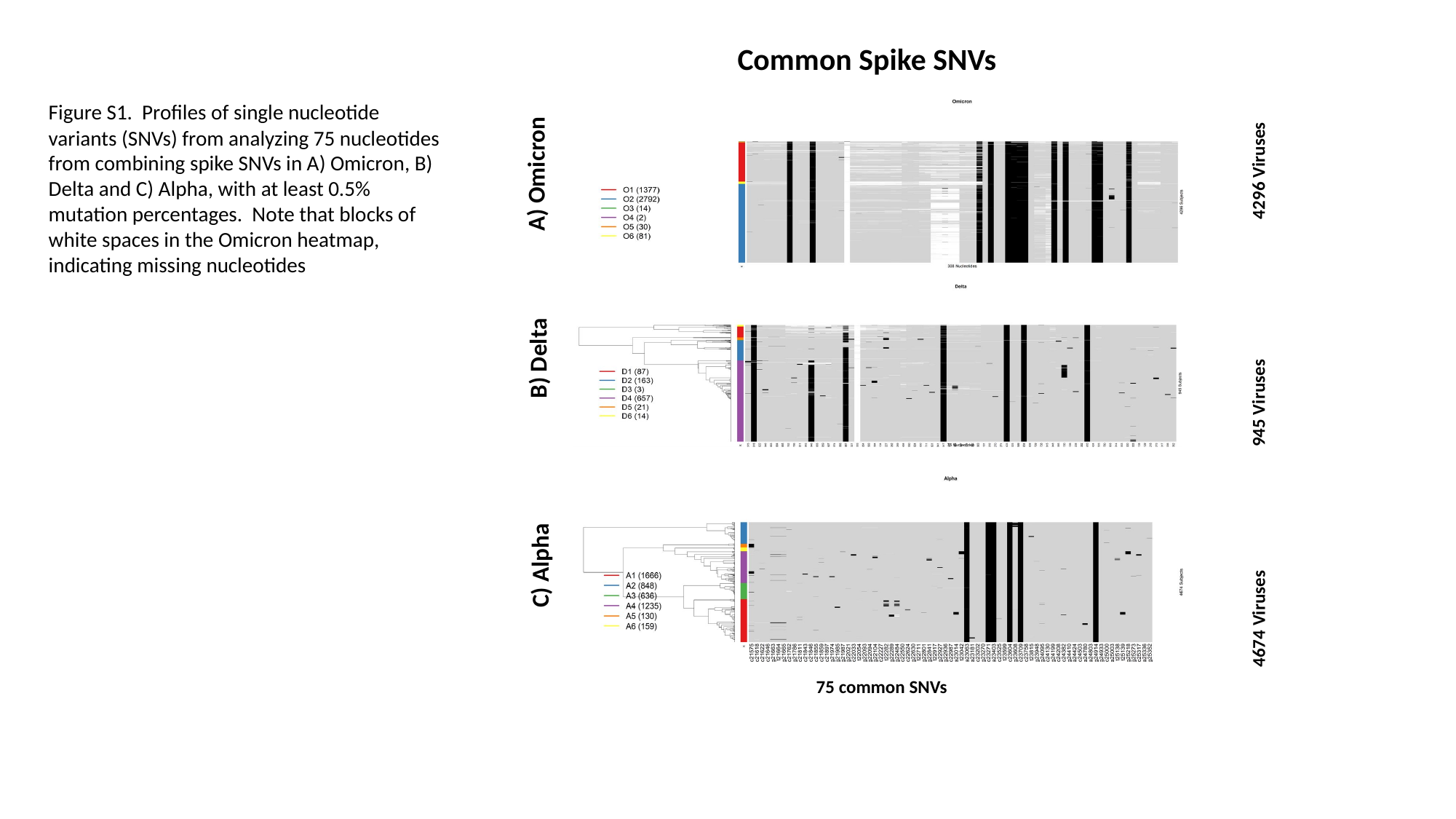

Common Spike SNVs
Figure S1. Profiles of single nucleotide variants (SNVs) from analyzing 75 nucleotides from combining spike SNVs in A) Omicron, B) Delta and C) Alpha, with at least 0.5% mutation percentages. Note that blocks of white spaces in the Omicron heatmap, indicating missing nucleotides
4296 Viruses
A) Omicron
B) Delta
945 Viruses
C) Alpha
4674 Viruses
75 common SNVs

## Slide 2
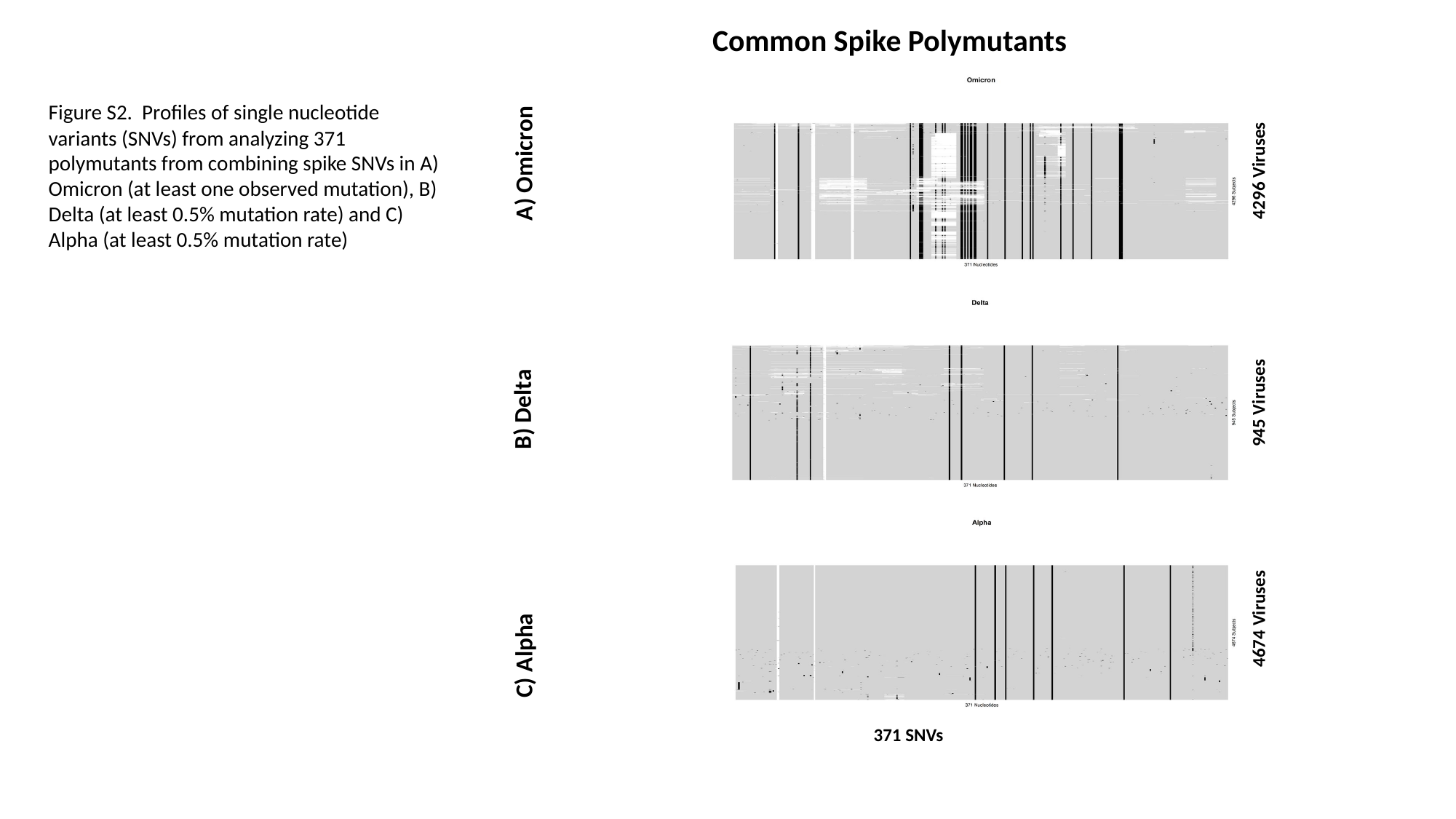

Common Spike Polymutants
Figure S2. Profiles of single nucleotide variants (SNVs) from analyzing 371 polymutants from combining spike SNVs in A) Omicron (at least one observed mutation), B) Delta (at least 0.5% mutation rate) and C) Alpha (at least 0.5% mutation rate)
A) Omicron
4296 Viruses
945 Viruses
B) Delta
4674 Viruses
C) Alpha
371 SNVs

## Slide 3
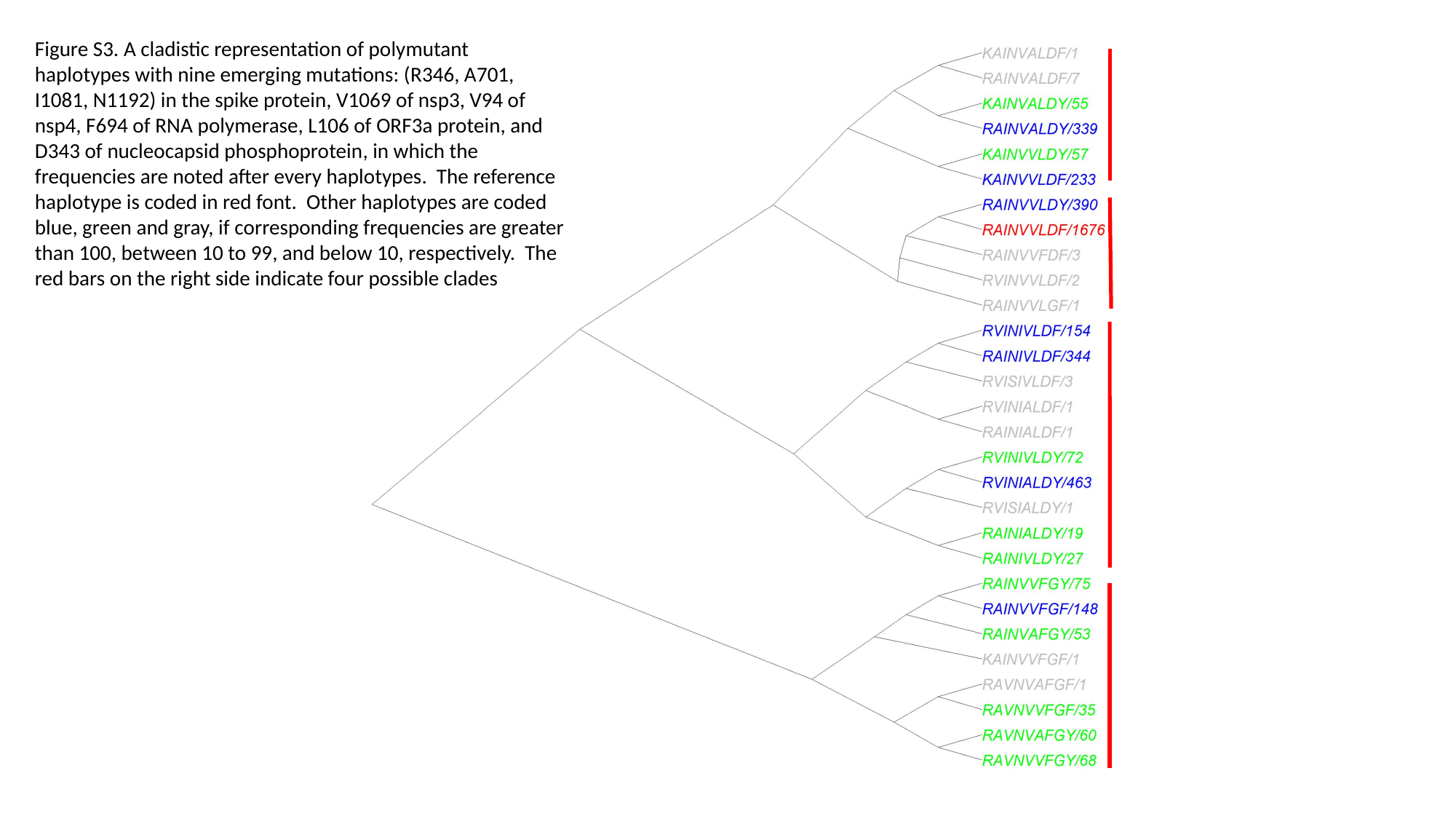

Figure S3. A cladistic representation of polymutant haplotypes with nine emerging mutations: (R346, A701, I1081, N1192) in the spike protein, V1069 of nsp3, V94 of nsp4, F694 of RNA polymerase, L106 of ORF3a protein, and D343 of nucleocapsid phosphoprotein, in which the frequencies are noted after every haplotypes. The reference haplotype is coded in red font. Other haplotypes are coded blue, green and gray, if corresponding frequencies are greater than 100, between 10 to 99, and below 10, respectively. The red bars on the right side indicate four possible clades
