# Supplementary material for: Rapidly Identifying New Coronavirus Mutations of Potential Concern in the Omicron Variant Using an Unsupervised Learning Strategy
Source: Res Sq. 2022 Feb 25:rs.3.rs-1280819. Preprint. [Version 1] doi: 10.21203/rs.3.rs-1280819/v1 (PMC8887078; doi:10.21203/rs.3.rs-1280819/v1)
Supplement: Supplement 2 [file 8852f1e2fa060178995a47b9.pdf]

Table S1. Descriptive table of SNV numbers by continent/country/region/sub-region around the
number, 1st quartile, median, mean, 3rd quartile and maximum number of mutations on each v

	Continent/Country/Region/Sub-region
1	Africa/Botswana/Gaborone
2	Africa/Botswana/Lobatse
3	Africa/Botswana/Otse
4	Africa/Botswana/Palapye
5	Africa/Botswana/South East/Greater Gaborone/Gaborone
6	Africa/Botswana/South East/Greater Gaborone/Lobatse
7	Africa/Botswana/South East/Greater Gaborone/Mochudi
8	Africa/Botswana/South East/Greater Gaborone/Oodi
9	Africa/Botswana/South East/Greater Gaborone/Ramotswa
10	Africa/Ghana/Accra
11	Africa/Malawi/Blantyre
12	Africa/Nigeria/Abuja
13	Africa/Reunion
14	Africa/Senegal/Dakar/Iressef Diamniadio
15	Africa/Senegal/Dakar/IRESSEF DIAMNIADIO
16	Africa/Sierra Leone
17	Africa/South Africa
18	Africa/South Africa/Eastern Cape
19	Africa/South Africa/Eastern Cape/Nelson Mandela Bay
20	Africa/South Africa/Eastern Cape/Sarah Baartman
21	Africa/South Africa/Free State
22	Africa/South Africa/Gauteng
23	Africa/South Africa/Gauteng/City of Johannesburg Metro
24	Africa/South Africa/Gauteng/City of Johannesburg Metro Region A
25	Africa/South Africa/Gauteng/City of Johannesburg Metro Region B
26	Africa/South Africa/Gauteng/City of Johannesburg Metro Region C
27	Africa/South Africa/Gauteng/City of Johannesburg Metro Region E
28	Africa/South Africa/Gauteng/City of Johannesburg Metro Region F
29	Africa/South Africa/Gauteng/Sedibeng
30	Africa/South Africa/Gauteng/Tshwane
31	Africa/South Africa/Kwazulu-Natal
32	Africa/South Africa/KwaZulu-Natal
33	Africa/South Africa/KwaZulu-Natal/eThekwini
34	Africa/South Africa/KwaZulu-Natal/Ilembe
35	Africa/South Africa/KwaZulu-Natal/King Cetshwayo
36	Africa/South Africa/KwaZulu-Natal/Ugu
37	Africa/South Africa/KwaZulu-Natal/uMkhanyakude
38	Africa/South Africa/KwaZulu-Natal/Zululand
39	Africa/South Africa/Limpopo
40	Africa/South Africa/Mpumalanga
41	Africa/South Africa/North West

42 Africa/South Africa/Northern Cape
43 Africa/South Africa/Western Cape
44 Africa/South Africa/Western Cape Province/Cape Town Metro
45 Asia/Bangladesh/Dhaka/Dhaka
46 Asia/Hong Kong
47 Asia/India/Delhi
48 Asia/India/Gujarat/Jamnagar
49 Asia/India/Karnataka
50 Asia/India/Maharashtra/Pune
51 Asia/India/New Delhi
52 Asia/Israel
53 Asia/Japan
54 Asia/Jordan/Amman
55 Asia/Malaysia/Selangor
56 Asia/Maldives/Sandies Bathala resort
57 Asia/Nepal/Bagmati
58 Asia/Pakistan
59 Asia/Singapore
60 Asia/South Korea
61 Asia/Sri Lanka/Marawila
62 Asia/Thailand/Bangkok/Bangrak
63 Asia/Thailand/Bangkok/Nongkhaem
64 Europe/Austria/Burgenland
65 Europe/Austria/Lower Austria
66 Europe/Austria/Lower Austria/Bruck an der Leitha/Schwechat
67 Europe/Austria/Tyrol/Schwaz
68 Europe/Austria/Tyrol/Schwaz/Vomp
69 Europe/Austria/Upper Austria/VÃ¶cklabruck/St. Georgen im Attergau
70 Europe/Austria/Vienna
71 Europe/Belgium
72 Europe/Belgium/Antwerpen
73 Europe/Belgium/Beersel
74 Europe/Belgium/Brabant Wallon
75 Europe/Belgium/Brussels
76 Europe/Belgium/Brussels Capital Region
77 Europe/Belgium/Flemish Brabant
78 Europe/Belgium/Geel
79 Europe/Belgium/Ghent
80 Europe/Belgium/Hainaut
81 Europe/Belgium/Jette
82 Europe/Belgium/Liege
83 Europe/Belgium/Limburg
84 Europe/Belgium/Meerhout
85 Europe/Belgium/Namur
86 Europe/Belgium/Putte
87 Europe/Belgium/Sint-agatha-berchem
88 Europe/Belgium/Sint-Agatha-Berchem

89 Europe/Belgium/Sint-Pieters-Leeuw
90 Europe/Belgium/Vlamertinge
91 Europe/Belgium/Wallonie/Hainaut/Enghien
92 Europe/Belgium/Wilrijk
93 Europe/Croatia/The City of Zagreb
94 Europe/Croatia/Zagreb County
95 Europe/Czech Republic/Liberec Region/Rynoltice
96 Europe/Czech Republic/South Moravian Region/Adamov
97 Europe/Czech Republic/South Moravian Region/Brno
98 Europe/Czech Republic/South Moravian Region/Slavkov u Brna
99 Europe/Denmark
100 Europe/Denmark/Hovedstaden
101 Europe/Denmark/Midtjylland
102 Europe/Denmark/Nordjylland
103 Europe/Denmark/Sjaelland
104 Europe/Denmark/Syddanmark
105 Europe/Finland
106 Europe/France/Auvergne-Rhone-Alpes/Clermont Ferrand
107 Europe/France/Auvergne-Rhone-Alpes/Haute-Savoie
108 Europe/France/Auvergne-Rhone-Alpes/Thonon les bains
109 Europe/France/Bretagne/Rennes
110 Europe/France/Centre-Val de Loire/Eure-et-Loir
111 Europe/France/Centre-Val de Loire/Luisant
112 Europe/France/Ile-de-France
113 Europe/France/Nouvelle-Aquitaine
114 Europe/France/Nouvelle-Aquitaine/Bordeaux
115 Europe/France/Pays de loire/La roche sur yon
116 Europe/France/Provence-Alpes-Cote d Azur
117 Europe/France/Provence-Alpes-Cote dâ€™Azur
118 Europe/France/Provence-Alpes-Cote dâ€™Azur/Alpes-Maritimes
119 Europe/Germany/Baden-Wurttemberg
120 Europe/Germany/Bavaria
121 Europe/Germany/Bavaria/Munich
122 Europe/Germany/Berlin
123 Europe/Germany/Berlin/Berlin
124 Europe/Germany/Brandenburg
125 Europe/Germany/Hamburg
126 Europe/Germany/Hesse
127 Europe/Germany/Lower Saxony
128 Europe/Germany/North Rhine-Westphalia
129 Europe/Germany/North Rhine-Westphalia/DÃ¼sseldorf Health department
130 Europe/Germany/Saxony
131 Europe/Germany/Schleswig-Holstein
132 Europe/Germany/Thuringia
133 Europe/Gibraltar
134 Europe/Greece
135 Europe/Ireland/Dublin

136 Europe/Ireland/Longford
137 Europe/Ireland/Meath
138 Europe/Italy/Calabria
139 Europe/Italy/Campania
140 Europe/Italy/Lombardia
141 Europe/Italy/Piemonte
142 Europe/Italy/Puglia
143 Europe/Italy/Trentino-Alto Adige
144 Europe/Liechtenstein
145 Europe/Netherlands/Gelderland
146 Europe/Netherlands/Noord-Holland
147 Europe/Netherlands/Noord-Holland/Amsterdam
148 Europe/Netherlands/Utrecht
149 Europe/Netherlands/Zeeland/Tholen
150 Europe/Norway/Oslo
151 Europe/Norway/Vestland
152 Europe/Norway/Viken
153 Europe/Portugal
154 Europe/Romania/Bucuresti
155 Europe/Russia/Moscow
156 Europe/Slovakia/Nitra
157 Europe/Slovakia/Presov
158 Europe/Spain/Albacete
159 Europe/Spain/Balearic Island
160 Europe/Spain/Basque Country/Donostia-San Sebastian
161 Europe/Spain/Castilla-La Mancha/Ciudad Real
162 Europe/Spain/Catalunya
163 Europe/Spain/Catalunya/Barcelona
164 Europe/Spain/Catalunya/Girona
165 Europe/Spain/Galicia/Vigo
166 Europe/Spain/Madrid
167 Europe/Sweden/Jonkopings lan
168 Europe/Sweden/Skane
169 Europe/Sweden/Stockholm
170 Europe/Switzerland/Aargau
171 Europe/Switzerland/Basel-Landschaft
172 Europe/Switzerland/Basel-Stadt
173 Europe/Switzerland/Bern
174 Europe/Switzerland/BL
175 Europe/Switzerland/BS
176 Europe/Switzerland/Geneva
177 Europe/Switzerland/Solothurn
178 Europe/Switzerland/Thurgau
179 Europe/Switzerland/Vaud
180 Europe/Switzerland/Zug
181 Europe/Switzerland/Zurich
182 Europe/Turkey

183 Europe/United Kingdom/England
184 Europe/United Kingdom/Scotland
185 Europe/United Kingdom/Wales
186 North America/Canada/Alberta
187 North America/Canada/British Columbia
188 North America/Canada/Manitoba
189 North America/Canada/Ontario
190 North America/Canada/Quebec
191 North America/Canada/Saskatchewan
192 North America/Mexico/Mexico City
193 North America/Mexico/State of Mexico
194 North America/Puerto Rico
195 North America/USA/Alaska/Anchorage-Mat Su
196 North America/USA/Arizona/Maricopa County
197 North America/USA/Arizona/Pima County
198 North America/USA/California
199 North America/USA/California/Alameda County
200 North America/USA/California/Los Angeles County
201 North America/USA/California/San Francisco County
202 North America/USA/California/West Sacramento
203 North America/USA/Colorado
204 North America/USA/Connecticut
205 North America/USA/Connecticut/Fairfield
206 North America/USA/Connecticut/Farifield
207 North America/USA/Connecticut/Hartford County
208 North America/USA/Connecticut/New Haven
209 North America/USA/District of Columbia
210 North America/USA/Florida
211 North America/USA/Georgia
212 North America/USA/Hawaii
213 North America/USA/Hawaii/Honolulu County
214 North America/USA/Idaho
215 North America/USA/Illinois/Cook County/Chicago
216 North America/USA/Iowa
217 North America/USA/Louisiana
218 North America/USA/Louisiana/Caddo Parish
219 North America/USA/Louisiana/Orleans
220 North America/USA/Louisiana/St Tammany
221 North America/USA/Maryland
222 North America/USA/Massachusetts
223 North America/USA/Massachusetts/Middlesex County
224 North America/USA/Michigan
225 North America/USA/Minnesota
226 North America/USA/Mississippi
227 North America/USA/Missouri
228 North America/USA/Nebraska
229 North America/USA/New Jersey

230	North America/USA/New York
231	North America/USA/New York/New York City
232	North America/USA/New York/Oneida
233	North America/USA/New York/Suffolk
234	North America/USA/New York/Westchester
235	North America/USA/Ohio
236	North America/USA/Oregon/Multnomah County
237	North America/USA/Oregon/Washington County
238	North America/USA/Pennsylvania
239	North America/USA/Rhode Island
240	North America/USA/Tennessee
241	North America/USA/Texas
242	North America/USA/Texas/Ellis
243	North America/USA/Texas/Houston
244	North America/USA/Texas/Houston County
245	North America/USA/Texas/San Antonio County
246	North America/USA/Texas/Tarrant County
247	North America/USA/Utah
248	North America/USA/Virginia
249	North America/USA/Washington
250	North America/USA/Washington/King County
251	North America/USA/Washington/Whatcom County
252	North America/USA/Wisconsin
253	North America/USA/Wisconsin/Milwaukee County
254	Oceania/Australia/Australian Capital Territory
255	Oceania/Australia/New South Wales/Sydney
256	Oceania/Australia/Northern Territory
257	Oceania/Australia/Queensland
258	South America/Argentina/San Luis
259	South America/Brazil/Distrito Federal/Brasilia
260	South America/Brazil/Federal District/Brasilia
261	South America/Brazil/Rio Grande do Sul/Santa Cruz do Sul
262	South America/Brazil/SÃ£o Paulo
263	South America/Brazil/Sao Paulo
264	South America/Brazil/Sao Paulo/Guarulhos
265	South America/Brazil/Sao Paulo/Limeira

world: number of reported viruses, minimum
iral genome

Freq	Min	1st	Median	Mean	3rd	Max
17	14	26	26	26.35	30	30
1	31	31	31	31.00	31	31
1	26	26	26	26.00	26	26
3	30	30	30	30.00	30	30
52	12	28	30	28.13	31	38
2	28	28.25	28.5	28.50	28.75	29
1	29	29	29	29.00	29	29
1	21	21	21	21.00	21	21
1	16	16	16	16.00	16	16
33	29	29	30	30.12	31	32
3	30	30.5	31	31.00	31.5	32
11	28	30	30	30.00	30.5	31
2	29	29.25	29.5	29.50	29.75	30
1	15	15	15	15.00	15	15
6	29	29.25	30.5	31.00	32.5	34
1	29	29	29	29.00	29	29
6	24	26	29.5	28.33	30.75	31
23	28	30	30	30.57	31	35
8	28	29	30	29.88	30	33
6	30	30.25	31	30.67	31	31
11	25	28.5	29	29.18	30	32
257	18	29	29	29.30	30	34
2	29	29.25	29.5	29.50	29.75	30
2	29	29	29	29.00	29	29
4	29	29	29	29.25	29.25	30
6	29	29	29.5	29.50	30	30
14	29	29	29.5	29.64	30	32
21	29	29	29	29.67	30	31
4	28	29.5	30.5	30.00	31	31
27	27	29	30	30.00	30	39
1	23	23	23	23.00	23	23
90	23	29	30	30.09	31	34
42	29	30	31	30.95	32	33
6	29	29.25	30	30.00	30.75	31
40	22	29	30	30.35	32	35
10	30	30	31	30.80	31	33
3	29	29.5	30	29.67	30	30
13	28	31	31	31.15	32	34
6	29	29.25	30	30.00	30.75	31
35	22	27.5	28	28.37	30.5	32
30	26	29	29.5	29.43	30.75	32

37	20	28	29	28.32	29	32
39	27	29	30	29.79	30	32
157	23	29	29	29.36	30	33
2	29	29.25	29.5	29.50	29.75	30
18	29	29	29.5	29.78	30.75	31
2	29	29.5	30	30.00	30.5	31
1	46	46	46	46.00	46	46
2	30	30	30	30.00	30	30
1	32	32	32	32.00	32	32
1	30	30	30	30.00	30	30
67	19	29	30	29.24	31	32
7	30	30	32	31.29	32	33
2	29	29.25	29.5	29.50	29.75	30
1	20	20	20	20.00	20	20
1	22	22	22	22.00	22	22
2	29	29	29	29.00	29	29
1	29	29	29	29.00	29	29
13	29	30	31	31.15	31	35
9	29	30	30	29.89	30	30
2	21	23.75	26.5	26.50	29.25	32
1	26	26	26	26.00	26	26
2	32	32.25	32.5	32.50	32.75	33
2	26	27	28	28.00	29	30
5	29	29	32	31.20	33	33
2	30	30	30	30.00	30	30
1	29	29	29	29.00	29	29
3	29	29	29	29.00	29	29
1	31	31	31	31.00	31	31
3	30	30	30	30.33	30.5	31
2	30	30	30	30.00	30	30
1	32	32	32	32.00	32	32
1	30	30	30	30.00	30	30
1	30	30	30	30.00	30	30
2	32	32	32	32.00	32	32
11	30	30	31	31.18	31.5	34
5	27	28	30	29.40	30	32
1	30	30	30	30.00	30	30
3	29	29.5	30	30.33	31	32
29	26	30	30	30.10	31	33
1	34	34	34	34.00	34	34
1	31	31	31	31.00	31	31
1	32	32	32	32.00	32	32
2	30	30.25	30.5	30.50	30.75	31
1	34	34	34	34.00	34	34
5	21	30	30	28.60	30	32
1	25	25	25	25.00	25	25
1	34	34	34	34.00	34	34

3	29	29	29	29.33	29.5	30
1	28	28	28	28.00	28	28
1	32	32	32	32.00	32	32
1	30	30	30	30.00	30	30
1	32	32	32	32.00	32	32
1	32	32	32	32.00	32	32
1	29	29	29	29.00	29	29
2	29	29.5	30	30.00	30.5	31
1	27	27	27	27.00	27	27
1	29	29	29	29.00	29	29
1	30	30	30	30.00	30	30
46	29	30	31	30.67	31	32
66	29	32	32	31.85	32	34
17	30	32	32	32.18	33	34
4	29	29.75	30.5	30.50	31.25	32
12	23	30	30	29.83	30	33
3	30	30	30	30.33	30.5	31
1	26	26	26	26.00	26	26
2	29	29	29	29.00	29	29
1	30	30	30	30.00	30	30
2	28	28.25	28.5	28.50	28.75	29
1	30	30	30	30.00	30	30
1	29	29	29	29.00	29	29
6	30	31	31	31.00	31	32
1	27	27	27	27.00	27	27
1	30	30	30	30.00	30	30
1	29	29	29	29.00	29	29
15	27	30	30	30.20	31	31
3	29	29.5	30	29.67	30	30
1	31	31	31	31.00	31	31
6	30	31	31.5	31.83	32	35
18	29	29.25	30	30.44	31.75	33
23	24	30	30	29.57	30	33
1	29	29	29	29.00	29	29
1	31	31	31	31.00	31	31
3	29	29	29	29.67	30	31
7	28	29	30	29.86	30.5	32
24	29	29	30	29.92	31	32
4	28	28.75	29.5	29.25	30	30
6	29	29	29.5	30.17	30.75	33
3	27	27	27	27.00	27	27
1	27	27	27	27.00	27	27
3	30	30	30	30.33	30.5	31
1	32	32	32	32.00	32	32
6	31	33	33	33.83	34.5	38
1	28	28	28	28.00	28	28
1	30	30	30	30.00	30	30

1	30	30	30	30.00	30	30
4	25	28.75	30	29.00	30.25	31
1	30	30	30	30.00	30	30
3	29	32	35	40.00	45.5	56
2	27	28	29	29.00	30	31
2	36	36.75	37.5	37.50	38.25	39
2	28	28	28	28.00	28	28
1	29	29	29	29.00	29	29
1	26	26	26	26.00	26	26
1	32	32	32	32.00	32	32
39	28	29	30	30.08	31	33
21	29	29	31	30.71	32	34
1	29	29	29	29.00	29	29
1	29	29	29	29.00	29	29
19	29	29	29	29.05	29	30
1	29	29	29	29.00	29	29
12	29	29	29	29.50	30	31
23	21	29	31	29.96	31	32
2	30	30.25	30.5	30.50	30.75	31
2	29	29	29	29.00	29	29
2	30	30	30	30.00	30	30
1	30	30	30	30.00	30	30
3	30	30.5	31	30.67	31	31
6	19	29.5	31	29.00	31	33
1	29	29	29	29.00	29	29
5	28	31	31	30.80	32	32
10	30	31	31	31.50	31.75	34
1	30	30	30	30.00	30	30
1	32	32	32	32.00	32	32
2	32	32.25	32.5	32.50	32.75	33
22	30	32	32.5	33.27	34.75	41
1	30	30	30	30.00	30	30
1	31	31	31	31.00	31	31
14	21	28.25	29.5	29.07	32	33
2	30	30.25	30.5	30.50	30.75	31
6	30	30	30.5	30.50	31	31
2	31	31	31	31.00	31	31
5	28	28	29	29.00	30	30
3	31	31	31	31.00	31	31
8	31	31	31	31.00	31	31
1	29	29	29	29.00	29	29
1	31	31	31	31.00	31	31
1	24	24	24	24.00	24	24
3	30	30	30	30.00	30	30
1	29	29	29	29.00	29	29
16	28	30	31	31.13	32	38
1	30	30	30	30.00	30	30

1700	27	32	33	33.50	35	43	
412	27	31	32	32.22	34	39	
9	31	32	33	33.00	34	36	
10	24	29.25	30.5	30.10	32.75	33	10
5	30	30	31	30.60	31	31	5
5	30	30	30	30.20	30	31	5
29	23	30	31	32.00	32	41	29
1	30	30	30	30.00	30	30	1
4	29	30.5	31.5	31.25	32.25	33	4
2	31	31	31	31.00	31	31	
1	32	32	32	32.00	32	32	
1	29	29	29	29.00	29	29	
1	24	24	24	24.00	24	24	1
5	31	31	31	34.00	35	42	5
1	33	33	33	33.00	33	33	1
4	30	30	30	30.25	30.25	31	4
4	26	29	30	29.00	30	30	4
2	30	30.5	31	31.00	31.5	32	2
1	32	32	32	32.00	32	32	1
1	31	31	31	31.00	31	31	1
2	26	27	28	28.00	29	30	2
3	24	27.5	31	28.67	31	31	3
4	30	30	31	31.00	32	32	4
1	30	30	30	30.00	30	30	1
1	30	30	30	30.00	30	30	1
2	30	30.25	30.5	30.50	30.75	31	2
1	23	23	23	23.00	23	23	1
2	30	30	30	30.00	30	30	2
4	29	29	30	30.25	31.25	32	4
2	30	30.5	31	31.00	31.5	32	2
12	30	30	30	30.50	31	32	12
1	30	30	30	30.00	30	30	1
1	30	30	30	30.00	30	30	1
1	31	31	31	31.00	31	31	1
2	29	29.75	30.5	30.50	31.25	32	2
1	31	31	31	31.00	31	31	1
1	32	32	32	32.00	32	32	1
2	27	27	27	27.00	27	27	2
10	27	30	30	29.80	30	31	10
16	29	29.75	30	29.94	30	32	16
3	30	30	30	30.00	30	30	3
1	31	31	31	31.00	31	31	1
2	30	30	30	30.00	30	30	2
1	29	29	29	29.00	29	29	1
1	30	30	30	30.00	30	30	1
6	30	31	31	30.83	31	31	6
2	29	29.5	30	30.00	30.5	31	2

5	29	30	30	30.00	30	31	5
10	28	29	30	29.70	30.75	31	10
2	23	24	25	25.00	26	27	2
2	25	26	27	27.00	28	29	2
1	31	31	31	31.00	31	31	1
4	30	30	31	31.00	32	32	4
1	32	32	32	32.00	32	32	1
2	30	30.25	30.5	30.50	30.75	31	2
2	28	28.75	29.5	29.50	30.25	31	2
1	30	30	30	30.00	30	30	1
1	23	23	23	23.00	23	23	1
4	31	31.75	32	31.75	32	32	4
1	32	32	32	32.00	32	32	1
6	29	32.25	33.5	32.83	34	35	6
25	32	32	33	33.44	34	44	25
2	34	34.5	35	35.00	35.5	36	2
1	26	26	26	26.00	26	26	1
4	28	29.5	30.5	30.00	31	31	4
1	30	30	30	30.00	30	30	1
7	29	30	30	30.71	32	32	7
1	30	30	30	30.00	30	30	1
1	32	32	32	32.00	32	32	1
1	32	32	32	32.00	32	32	1
4	24	28.5	30	29.25	30.75	33	4
10	29	30	30	30.00	30	31	193
79	19	28	30	28.76	30	32	
1	29	29	29	29.00	29	29	
2	33	34.75	36.5	36.50	38.25	40	
1	30	30	30	30.00	30	30	
1	31	31	31	31.00	31	31	
1	30	30	30	30.00	30	30	
1	30	30	30	30.00	30	30	
1	30	30	30	30.00	30	30	
2	29	29	29	29.00	29	29	
1	30	30	30	30.00	30	30	
1	30	30	30	30.00	30	30	
